# Genetic association of stomatal traits and yield in wheat grown in low rainfall environments

**DOI:** 10.1186/s12870-016-0838-9

**Published:** 2016-07-04

**Authors:** Fahimeh Shahinnia, Julien Le Roy, Benjamin Laborde, Beata Sznajder, Priyanka Kalambettu, Saba Mahjourimajd, Joanne Tilbrook, Delphine Fleury

**Affiliations:** Present Address: Leibniz Institute of Plant Genetics and Crop Plant Research (IPK), 06466 Gatersleben, Germany; Australian Centre for Plant Functional Genomics and School of Agriculture, Food and Wine, Waite Research Institute, The University of Adelaide, PMB 1, Glen Osmond, South Australia 5064 Australia; University of Lille, CNRS, INRA, UMR 8576 - Unité de Glycobiologie Structurale et Fonctionnelle, 59000 Lille, France; Montpellier SupAgro, 2 place Viala, 34060 Montpellier Cedex 2, France

**Keywords:** Drought, Epidermal cells, Flag leaf, QTL, Stomatal density, Stomatal size, *Triticum aestivum*

## Abstract

**Background:**

In wheat, grain filling is closely related to flag leaf characteristics and function. Stomata are specialized leaf epidermal cells which regulate photosynthetic CO_2_ uptake and water loss by transpiration. Understanding the mechanisms controlling stomatal size, and their opening under drought, is critical to reduce plant water loss and maintain a high photosynthetic rate which ultimately leads to elevated yield. We applied a leaf imprinting method for rapid and non-destructive phenotyping to explore genetic variation and identify quantitative traits loci (QTL) for stomatal traits in wheat grown under greenhouse and field conditions.

**Results:**

The genetics of stomatal traits on the adaxial surface of the flag leaf was investigated using 146 double haploid lines derived from a cross between two Australian lines of *Triticum aestivum*, RAC875 and Kukri. The drought tolerant line RAC875 showed numerous small stomata in contrast to Kukri. Significant differences between the lines were observed for stomatal densitity and size related traits. A negative correlation was found between stomatal size and density, reflecting a compensatory relationship between these traits to maintain total pore area per unit leaf surface area. QTL were identified for stomatal traits on chromosomes 1A, 1B, 2B, and 7A under field and controlled conditions. Most importantly some of these loci overlap with QTL on chromosome 7A that control kernel number per spike, normalized difference vegetation index, harvest index and yield in the same population.

**Conclusions:**

In this first study to decifer genetic relationships between wheat stomatal traits and yield in response to water deficit, no significant correlations were observed among yield and stomatal traits under field conditions. However we found some overlaps between QTL for stomatal traits and yield across environments. This suggested that stomatal traits could be an underlying mechanism increasing yield at specific loci and used as a proxy to track a target QTL in recombinant lines. This finding is a step-forward in understanding the function of these loci and identifying candidate genes to accelerate positional cloning of yield QTL in wheat under drought.

**Electronic supplementary material:**

The online version of this article (doi:10.1186/s12870-016-0838-9) contains supplementary material, which is available to authorized users.

## Background

Cereal production will need to increase by 37 % to meet the food security challenge by 2050 [1]. Bread wheat (*Triticum aestivum* L.) is one of the most widely grown cereals and an essential component of the global food security, supplying one-fifth of the total calories of the world’s population [2]. Drought is a major abiotic stress that reduces wheat yield and production in the world. As a result of climate change, the global frequency and severity of drought events is likely to increase. For example, regional projections show that South-Eastern Australia will be affected by changes in rainfall patterns and rising temperatures with 40 % more months of drought in the region by 2070 [3]. A way to improve the drought tolerance of crops is to discover new genes and alleles that allow plants to continue to grow and maintain or increase grain yield under water-limited growing conditions.

Flag leaf is one of the major contributors to wheat grain yield, particularly under drought [4–7]. This is because of role of the flag leaf in the photosynthetic source-sink relationship, carbohydrate synthesis, accumulation and partitioning [7]. Restriction of water loss from the leaf during periods of severe water stress is an important survival mechanism. However, early stomatal closure decreases net photosynthesis by reducing photosynthetic activity of PSII, amounts of C fixed and activity of key photosynthetic enzymes resulting in a decrease in leaf area, leaf width and mean area per mesophyll cell and eventually losses in grain yield [7].

Stomatal and epidermal cells play an important role in the control of water evaporation and gas exchange in leaf [8, 9]. Stomata consist of two specialised guard cells which regulate CO_2_ uptake and transpiration by changing the size of stomatal pores [10]. Although the total stomatal pore area is 5 % of the leaf surface, transpirational water loss through the stomatal pores contributes to 70 % of total water use by plants [8]. Therefore, one of the important aspects in wheat breeding for increasing drought tolerance lies in a better understanding of the molecular mechanisms and genetic control of stomatal distribution and opening associated with growth rate and grain yield under abiotic stress [11, 12].

Depending on the environmental conditions and the species, stomatal size ranges between 10 and 80 μm in length with densities between 5 and 1000/mm^2^ of epidermis [8]. There is a strong negative relationship between stomatal density and size in all plant taxa [8, 13]. Larger stomata are usually distributed in low densities [13, 14]. Arabidopsis mutants with low stomatal density and large stomatal size showed reduced transpiration, larger biomass and an improved growth rate under water-limited conditions compared to wild-type [15].

Stomatal traits such as density and size are considered key determinants of growth rate and water balance in plants [14]. The distribution and frequency of stomata are coordinated with cell growth and division: signalling among cell types affects asymmetric division, cell-fate specification, as well as the establishment and maintenance of undifferentiated or stem-cell populations [15]. This phenomenon preserves a level of plasticity in response to ever-changing environmental conditions such as light, temperature and vapour pressure deficit. Stomatal traits are strongly controlled by genetic factors [16] with at least 40 genes known in Arabidopsis for regulating stomatal development [15]. An estimation of the number and effect of genes involved in stomatal traits in non-model species can be obtained by quantitative trait loci (QTL) analysis. QTL analysis has already been used to identify the genes underlying naturally occurring variation of stomatal traits in barley and rice [17, 18].

The objectives of this study were to: *(i)* evaluate the genetic variation of stomatal frequency and size related traits *(ii)* identify QTL controlling stomatal traits and yield and *(iii)* determine the genetic relationships among those traits in response to drought using a doubled-haploid (DH) mapping population derived from two Australian wheat lines RAC875 and Kukri.

## Results

### Phenotypic variations, heritability and correlation among the stomatal and yield traits

**Fig. 1:**
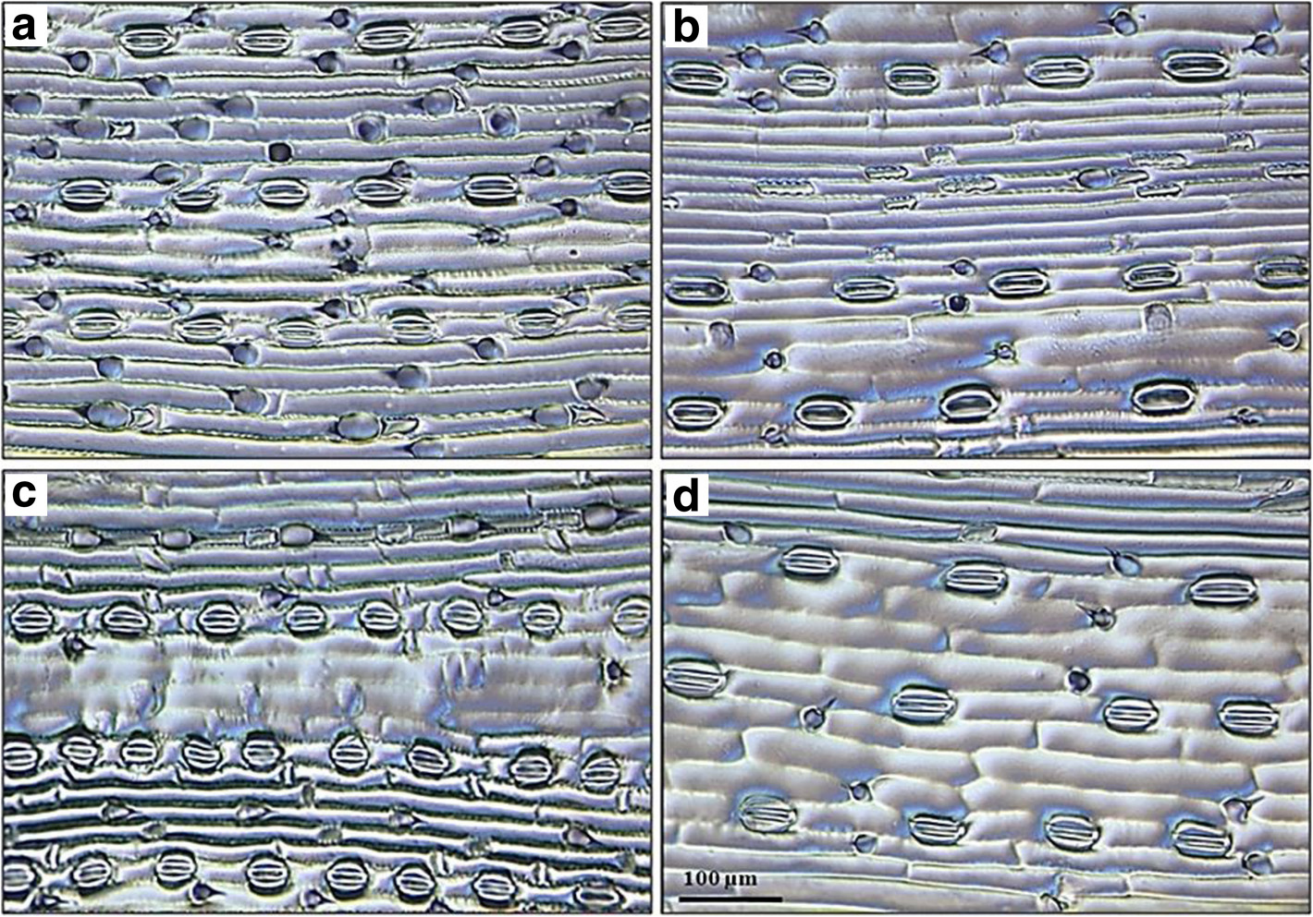
Leaf imprinting obtained from adaxial surface of the flag leaf. A comparison of the stomatal density and size between **a** RAC875 and **b** Kukri parental lines and **c** DH 214 and **d** DH 79 contrasting lines. The scale bar is 100 μm

The RAC875 parental line had significantly more stomata (1.05–1.35 times), of smaller size (10–20 %) and showed higher yield (5–14 %) than Kukri (Table 1; Additional file 1: Figure S1). Frequency distribution of the phenotypes showed a large continuous variation and transgressive segregation among the DH lines for stomatal traits and yield (Additional file 1: Figure S1). Two contrasting DH lines, DH 214 (Fig. 1c) and DH 79 (Fig. 1d), were identified for stomatal density (SD) and aperture area (APA) under drought treatment in the glasshouse: DH 214 showed high density of small stomata with an average across all experiments of 78.54 stomata/μm^2^ leaf area, and 168.42 μm^2^ average size of aperture area; DH 79 had large stomata in low density with an average of 47.40 stomata/μm^2^ leaf area, and 225.09 μm^2^ average size of aperture area.

**Table 1:** Parental values, descriptive statistics and ANOVA for stomatal traits and yield

Experiment	Trait	Stomatal Density	Stomatal index	Aperture length	Aperture width	Aperture area	Guard cell length	Guard cell width	Guard cell area	APL/APW	GCL/GCW	Yield
	Acronym	SD (n/mm^2^)	SI (%)	APL (μm)	APW (μm)	APA (μm^2^)	GCL (μm)	GCW (μm)	GCA (μm^2^)			YLD (t/ha)
Lameroo	Mean ± STD	63 ± 2.64	8.05 ± 1.12	31.31 ± 2.66	3.87 ± 0.44	121.39 ± 17.79	45.81 ± 3.12	10.01 ± 0.67	459.10 ± 49.31	8.21 ± 0.46	3.04 ± 0.67	2.32 ± 0.36
	Min-Max	43–84	5.45–11.21	23.71–38.03	1.68–4.80	77.5–174.51	37.74–54.72	8.08–11.90	343.20–626	6.19–16.99	3.4–5.68	1.39–3.25
	*h* ^*2*^ (%)	40	44	41	35	39	43	36	40	35	47	35
	*F*-test	**	**	**	ns	*	**	ns	**	ns	ns	*
	RAC875	71	10.82	30.30	3.68	111.49	43.46	9.47	411.35	8.23	4.58	2.31
	Kukri	69	9.44	30.43	4.21	127.94	43.96	10.79	474.33	7.22	4.07	2.18
Roseworthy	Mean ± STD	70 ± 1.80	9.98 ± 1.28	31.56 ± 2.28	4.89 ± 0.37	155.04 ± 20.84	44.92 ± 2.34	8.06 ± 0.45	386.91 ± 36.11	6.45 ± 0.32	5.22 ± 0.41	2.93 ± 0.28
	Min-Max	46–92	7.25–12.75	28.25–36.25	3.40–6.5	119.20–201.01	41.00–51.39	7.99–10.12	200.41–520.30	5.19–7.15	4.62–5.76	2.44–3.44
	*h* ^*2*^ (%)	50	42	34	34	33	36	34	33	46	36	43
	*F*-test	**	**	*	ns	*	*	ns	*	ns	ns	*
	RAC875	67	9.39	33.07	4.68	149.3	47.15	9.13	430.85	7.06	5.16	3.09
	Kukri	61	8.70	32.43	5.77	163.7	45.11	10.34	466.84	5.62	4.36	2.73
WW	Mean ± STD	65 ± 1.26	11.04 ± 1.26	37.77 ± 1.81	6.14 ± 0.96	232.88 ± 38.07	49.13 ± 2.17	12.27 ± 1.23	409.68 ± 55.35	6.26 ± 0.14	4.41 ± 0.23	-
	Min-Max	52–80	10.75–16.00	33.74–41.67	4.74–8.74	170.92–331.42	45.11–53.15	9.44–15.11	227.68–614.83	4.28–7.97	3.26–5.39	-
	*h* ^*2*^ (%)	42	42	39	39	38	41	39	40	39	41	-
	*F*-test	**	**	**	**	*	**	*	**	ns	ns	-
	RAC875	75	14	34.53	5.33	184.04	45.48	10.12	344.36	6.47	4.49	-
	Kukri	50	11	41.83	7.07	295.13	53.94	12.35	475.97	5.91	4.36	-
D	Mean ± STD	67 ± 1.83	10.84 ± 1.38	36.85 ± 2.30	5.04 ± 0.52	185.78 ± 22.81	48.44 ± 2.64	9.87 ± 0.7	360.31 ± 38.43	7.38 ± 0.64	4.92 ± 0.31	-
	Min-Max	53–89	11.25–17.14	30.68–42.48	4.16–6.63	142.08–244.43	41.16–55.15	8.40–11.85	227.31–436.57	4.98–8.97	3.74–6.11	-
	*h* ^*2*^ (%)	44	40	42	36	39	41	35	36	38	40	-
	*F*-test	**	**	**	ns	*	**	ns	**	ns	ns	-
	RAC875	80	16.83	33.18	5.09	166.20	44.03	9.87	324.83	6.51	4.46	-
	Kukri	57	11.18	40.51	5.37	214.78	52.27	9.81	373.37	7.54	5.32	-

Mean, standard deviation (STD), minimum (Min), maximum (Max), heritability (*h* ^*2*^) and significance of the variance between RAC875/Kukri DH lines (*F*-test) for the traits phenotyped under field (Lameroo, Roseworthy), well-watered (WW) and drought (D) glasshouse conditions. **, * and ns are *p* < 0.01, *p* < 0.05 and not-significant, respectively

Analysis of variance (Table 1) indicated significant differences among the lines for yield and most of the stomatal traits such as stomatal density and index (SI), aperture length (APL), guard cell length (GCL) and area (GCA) measured on the adaxial surface of the flag leaf. No significant difference was observed among DH lines for the ratio aperture length to aperture width (APL/APW) and guard cell length to guard cell width (GCL/GCW) in all environments tested. The broad sense heritability (*h*^*2*^) estimated from the components of variance for all of the traits ranged between 33 and 50 %, indicating that the proportion of genetic to environmental variation of each trait is low to medium in this population (Table 1). Traits showing significant differences between lines were used for QTL analysis. Analysis of variance (ANOVA) also showed highly significant differences (*p* < 0.01) among the lines for all of the traits under well-watered *versus* drought treatments in glasshouse, and Lameroo *versus* Roseworthy conditions in the field (Additional file 2: Table S1), indicating a strong effect of water stress on stomatal traits and yield.

**Fig. 2:**
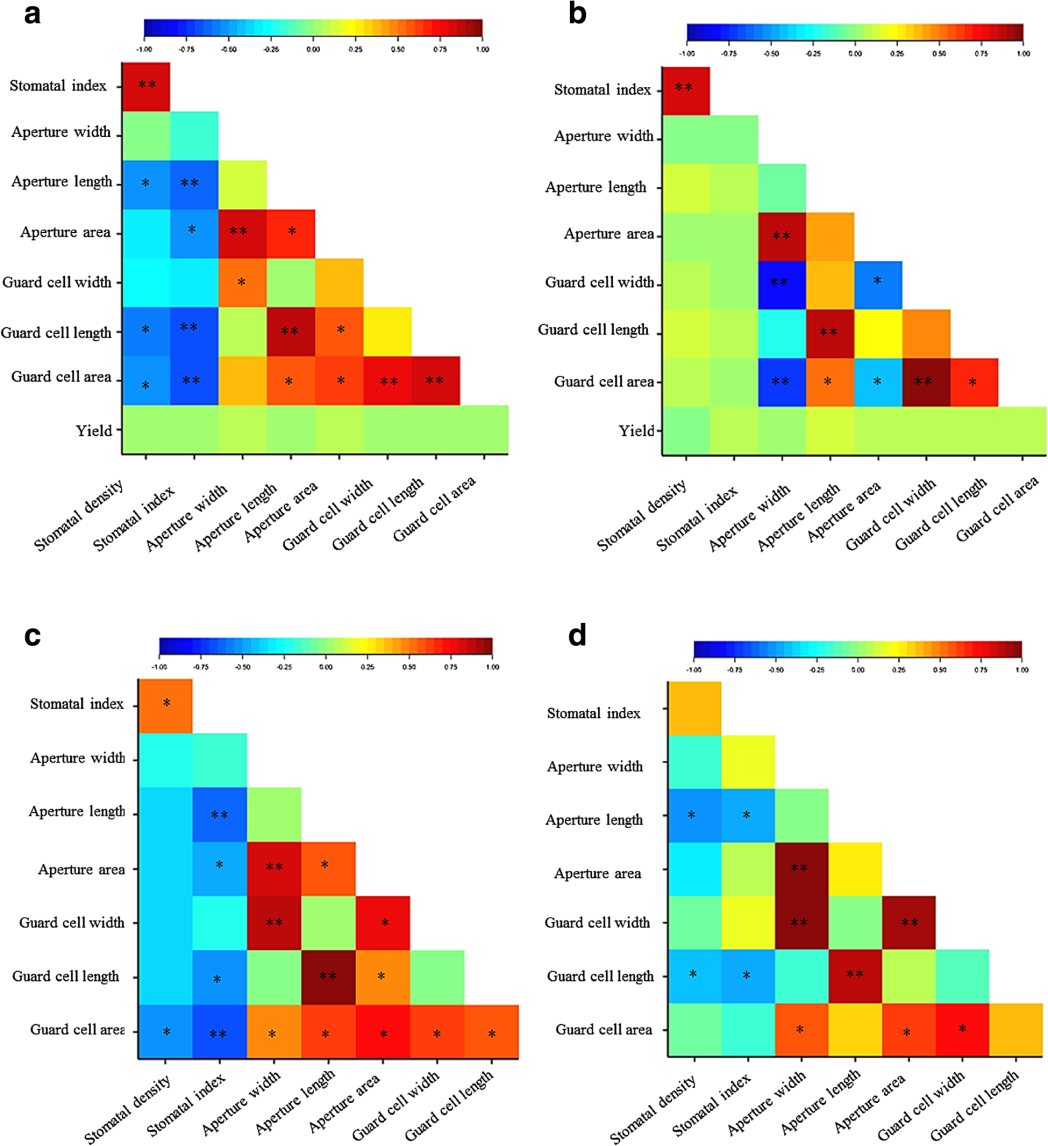
Phenotypic correlations among all the traits. Heat maps illustrating the correlations measured in the RAC875/Kukri DH population grown under **a** Lameroo and **b** Roseworthy field-conditions and **c** drought and **d** well-watered treatments in the glasshouse. According to colour key, correlation coefficients between r ≤ -0.3 and 0.3 ≤ r are significant at *p* < 0.01 (**) and *p* < 0.05 (*)

Similar correlations were observed among traits from the Lameroo field trial and the drought treatment in the glasshouse (Fig. 2) where most stomatal size related traits such as aperture and guard cell length and width were significantly and positively correlated to aperture area. Highly significant negative correlations were observed between stomatal density and index *versus* aperture and guard cell lengths in all experiments. No significant correlations between stomatal traits and yield were detected in the field.

### QTL mapping for stomatal traits under field-conditions

The analysis detected three QTL on chromosome arms 4AS and 5AS and linkage group 7A1L for SD in the Roseworthy and Lameroo field trials (Table 2). A strong QTL on 5AS (*QSD.afr-5A*) explained the highest phenotypic variance (18 %) of SD with the largest LOD score at Roseworthy with the RAC875 allele increasing the trait values. The QTL *QSI.afr-2B* and *QSI.afr-7B* were found for SI on 2BL and 7BL, explaining 32 % of the total phenotypic variation in Roseworthy.

**Table 2:** Quantitative trait loci for stomatal traits and yield

Trait/experiment	QTL symbol	Chr arm	Closest marker	Peak (cM)	LOD	AE	R2 (%)
Lameroo							
SD	*QSD.afl-7A*	7A1L	wsnp_Ra_rep_c104968_8898575	79.1	5.6	1.4	10
APL	*QAPL. afl-2B*	2BS	wsnp_Ra_c18396_27453775	42.41	5.4	1.0	10
	*QAPL. afl-7A*	7A1L	BobWhite_rep_c49790_351	85.2	5.2	−1.0	5
GCL	*QGCL. afl-7A*	7A1L	CAP8_c3496_118	83.7	6.5	−1.2	14
	*QGCL. afl-3B*	3BL	adli16	122.18	4.3	−0.2	9
GCA	*QGCA. afl-1B*	1BL	wPt.0944	158.44	3.6	14.9	8
	*QGCA. afl-4B*	4BL	BS00023024_51	86.2	3.5	13.8	6
	*QGCA. afl-5D*	5DL	RAC875_c41914_613	188.12	3.5	14.3	8
YLD	*QYLD. afl-4A*	4AS	BS00022177_51	14.9	9.0	0.17	23
Roseworthy							
SD	*QSD.afr-4A*	4AS	BS00011060_51	36.0	3.6	−0.6	8
	*QSD. afr-5A*	5AS	TA003745.0632	7.1	6.4	0.8	18
SI	*QSI. afr-2B*	2BL	Kukri_c657_1139	84.2	3.1	0.5	17
	*QSI. afr-7B*	7BL	BobWhite_c33540_69	64.4	3.1	0.5	15
APL	*QAPL. afr-2B*	2BL	wPt.3378	94.75	2.9	−0.7	9
	*QAPL. afr-7A*	7A1L	BobWhite_c1201_384	98.73	2.8	0.7	9
APA	*QAPA. afr-7A*	7A1L	BobWhite_c1201_384	98.73	4.5	−7.6	14
GCL	*QGCL. afr-1A*	1AS	wsnp_Ku_c34659_43981982	35.02	4.1	0.9	13
	*QGCL. afr-7A*	7A1L	BobWhite_c1201_384	95.5	3.5	0.1	8
GCA	*QGCA. afr-5A*	5AL	Kukri_c28080_887	109.9	3.9	−7.2	6
YLD	*QYLD. afr-7A*	7A1L	BobWhite_c15497_199	84.43	4.53	0.24	16
	*QYLD. afr-7B*	7BS	RAC875_c7123_1703	46.45	3.0	0.39	9
WW							
SD	*QSD.atw-3A*	3A2L	BobWhite_c43681_334	90.9	6.7	0.7	26
	*QSD.atw-4B*	4BS	tplb0061a20_153	67.3	7.8	0.7	28
	*QSD.atw-7A*	7A1S	Excalibur_c15260_94	51.2	4.2	0.5	14
SI	*QSI.atw-3D*	3D2S	Excalibur_c3510_1888	19.67	4.5	−6.3	23
APL	*QAPL.atw-1A*	1AS	Tdurum_contig9199_714	39.15	4.9	1.3	13
	*QAPL.atw-4B*	4BS	BS00037094_51	21.7	5.9	−0.9	19
APW	*QAPW.atw-1B*	1BL	barc0207	116.56	6.0	0.9	22
	*QAPW.atw-7A*	7A1L	BobWhite_c23146_84	76.0	6.6	−0.5	22
	*QAPW.atw-2D*	2DS	Ex_c10377_845	60.85	12.6	0.7	22
APA	*QAPA.atw-2D*	2DS	Ex_c10377_845	60.85	14.6	31.7	53
	*QAPA.atw-7A*	7A1L	BobWhite_c23146_84	76.0	5.8	−18.5	18
GCL	*QGCL.atw-1B*	1BL	barc0207	116.56	5.7	1.5	23
	*QGCL.atw-7D*	7DL	BS00067285_51	113.0	4.0	0.9	15
GCW	*QGCW.atw-2D*	2DS	Ex_c10377_845	60.85	4.0	0.5	13
	*QGCW.atw-6A*	6AS	BS00061749_51	21.97	4.1	0.5	14
GCA	*QGCA.atw-4A*	4AL	BS00022395_51	133.2	3.3	20.0	12
	*QGCA.atw-4B*	4BS	Kukri_c26488_139	18.8	5.0	27.0	19
D							
SD	*QSD.atd-5B*	5BS	Excalibur_c29975_333	28.6	5.2	0.9	19
SI	*QSI.atd-5B*	5BS	Excalibur_c17904_437	34.3	4.8	6.1	19
	*QSI.atd-6D*	6DL	RFL_Contig1159_2123	106.22	5.2	6.2	18
APL	*QAPL.atd-2B*	2BL	Excalibur_c15242_306	87.39	3.0	−1.0	14
	*QAPL.atd-4B*	4BS	BS00037094_51	22.8	4.0	−0.8	11
	*QAPL.atd-7A*	7A1S	Kukri_c4700_1266	1.7	3.7	−0.8	10
	*QAPL.atd-7D*	7DL	BS00067285_51	129.3	5.0	−0.4	17
GCL	*QGCL.atd-1B*	1BL	wPt.0944	158.44	3.4	1.4	15
	*QGCL.atd-4B*	4BS	Excalibur_c7581_1266	24.6	6.0	−1.3	26
	*QGCL.atd-7A*	7A1S	BS00081098_51	4.6	6.0	−1.4	26
GCA	*QGCA.atd-1B*	1BL	wsnp_Ra_c21132_30487331	112.91	4.0	17.4	17
	*QGCA.atd-5B*	5BS	Excalibur_c29975_333	28.5	6.5	−25.0	32

Chromosomal location, closest linked marker, position of the QTL peak, LOD score, estimated additive effects (AE, in trait unit) and percentage of phenotypic variance (R^2^) for QTL assessed in RAK875/Kukri DH population in the field (Lameroo and Roseworthy), and under well-watered (WW) and drought (D) glasshouse conditions. A positive AE value means the trait increased is due to RAC875 allele at the QTL. APA: aperture area, APL: aperture length, APW: aperture width, GCA: guard cell area, GCL: guard cell length, GCW: guard cell width, SD: stomatal density, SI: stomatal index, YLD: yield

Five QTL on chromosome arms 2BS, 2BL and linkage group 7A1L were identified for APL and APA under field conditions (Table 2). Of those, the QTL *QAPL.afl-2B* on 2BS and *QAPA.afl-7A* on 7A1L explained 10 and 14 % of the phenotypic variation found in Lameroo and Roseworthy respectively, of APL and APA. The QTL *QAPA.afr-7A* near BobWhite_c1201_384 marker showed the highest negative additive effect indicating that the Kukri allele increases APA values.

Four QTL were detected for GCL on 3BL and 7A1L in Lameroo and on 1AS and 7A1L in Roseworthy (Table 2). The QTL *QGCL.afl-7A* on 7A1L explained the highest phenotypic variation of GCL in Lameroo. The allele carried by RAC875 for this QTL decreased GCL. The 22 % total phenotypic variation of GCA was explained by the QTL *QGCA.afl-1B*, *QGCA.afl-4B* and *QGCA.afl-5D* which were identified on 1BL, 4BL and 5DL respectively, in Lameroo, with a positive additive effect of RAC875 at all loci.

### Stability of QTL for stomatal traits across environments

In order to find the conditions that control some of the QTL for stomatal traits, we investigated whether the QTL identified in the field could also be found in plants grown in pots under controlled conditions using a small set of DH lines segregating for the QTL. Although using a small number of lines is not ideal to find *de novo* QTL, we found a total of 29 QTL. Of those, seven QTL were identified for SD and SI in the glasshouse, including four QTL under well-watered and three under drought conditions (Table 2). Seven QTL controlled aperture characteristics on 1AS, 1BL, 2DS, 2DL, 4BS and 7A1L in the well-watered treatment, while four QTL were identified only for aperture length on 2BL, 4BS, 7A1S and 7DL under drought conditions (Table 2). Eleven QTL for guard cell size were detected on 1BL, 2DS, 4BS, 4AL, 5BS, 6AS, 7A1S and 7DL in well-watered and drought conditions in the glasshouse (Table 2).

Co-located QTL for stomatal traits were found in field and controlled conditions on chromosome arms 1AS, 1BL, 2BL and 7A1L (Table 3). The *QGCL.afr-1A* QTL for guard cell length from Roseworthy trial overlapped with the QTL for aperture length *QAPL.atw-1A* under well-watered controlled conditions. Both traits, guard cell and aperture length, were closely related to one another as shown by the positive correlation ranging from 0.75 and 0.95 (Fig. 2). The RAC875 allele at these QTL increased the guard cell length by 0.9 μm in field and the aperture length by 1.3 μm in the glasshouse (Table 2).

**Table 3:**
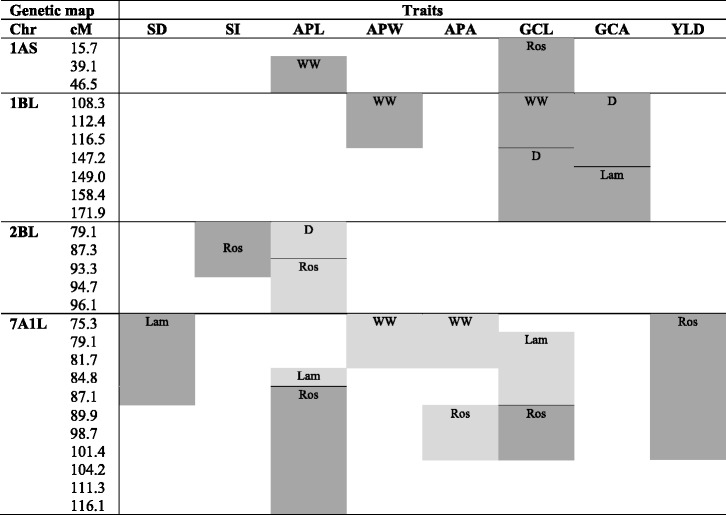
Co-location of QTL detected for yield in field and stomatal traits in field- and controlled-conditions

Chromosomal location and genetic position (cM) of overlapped QTL in RAC875/Kukri DH population in Lameroo (Lam) and Roseworthy (Ros) trials, and under well-watered (WW) and drought (D) glasshouse conditions. Positive and negative additive allelic effect of the RAC875 parental line is shown with dark and light grey, respectively. APA: aperture area, APL: aperture length, APW: aperture width, GCA: guard cell area, GCL: guard cell length, SD: stomatal density, SI: stomatal index, YLD: yield

On chromosome arm 1BL, four overlapping QTL, *QGCA.afl-1B*, *QGCL.atd-1B*, *QAPW.atw-1B* and *QGCL.atd-1B*, were identified for controlling stomatal and aperture size at Lameroo and under well-watered and drought in the glasshouse. The RAC875 alleles showed positive additive effect on all of these loci increasing the guard cell area by 14.9 μm^2^ in field and 17.4 μm^2^ under drought in controlled conditions.

On chromosome arm 2BL, three QTL, *QSI.afr-2B*, *QAPL.afr-2B* and *QAPL.atd-2B*, overlap between the 84.2 and 94.75 cM region of the long arm. The Kukri allele increased APL by 0.7 μm in Roseworthy field conditions and 1.0 μm under drought treatment in the glasshouse while decreasing stomatal index by 0.5 % in the field (Table 2).

A total of six QTL were identified on chromosome arm 7A1L for stomatal traits in Lameroo and Roseworthy field trials, and two QTL in well-watered controlled conditions. The QTL overlap in the interval between 75.3 and 84.8 cM suggested the Kukri allele increased APL and GCL while decreasing SI in Lameroo, and increased APW and APA in the glasshouse. The QTL found in the Roseworthy trial, *QAPL.afr-7A*, *QAPA.afr-7A* and *QGCL.afr-7A*, located in the interval 87.1 to 116.1 cM showed positive additive effects for APL and GCL, meaning RAC875 allele increases APL and GCL. The opposite additive effects suggest that these QTL are different than those found at Lameroo and the glasshouse.

### Co-location between QTL controlling stomatal traits and yield

A total of three QTL for yield (*QYLD.afl-4A*, *QYLD.afr-7A* and *QYLD.afr-7B*) were identified under field-conditions. A strong yield QTL (*QYLD.afl-4A*) was found on chromosome arm 4AS and explained 23 % of the variation for the trait in Lameroo (Table 2). The effect of RAC875 allele in this locus increased yield by 0.17 t/ha. Two more QTL for yield, *QYLD.afr-7A* and *QYLD.afr-7B*, were detected on 7A1L and 7BS in Roseworthy, explaining 25 % of the total phenotypic variation with positive additive effect of RAC875 for both loci.

**Fig. 3:**
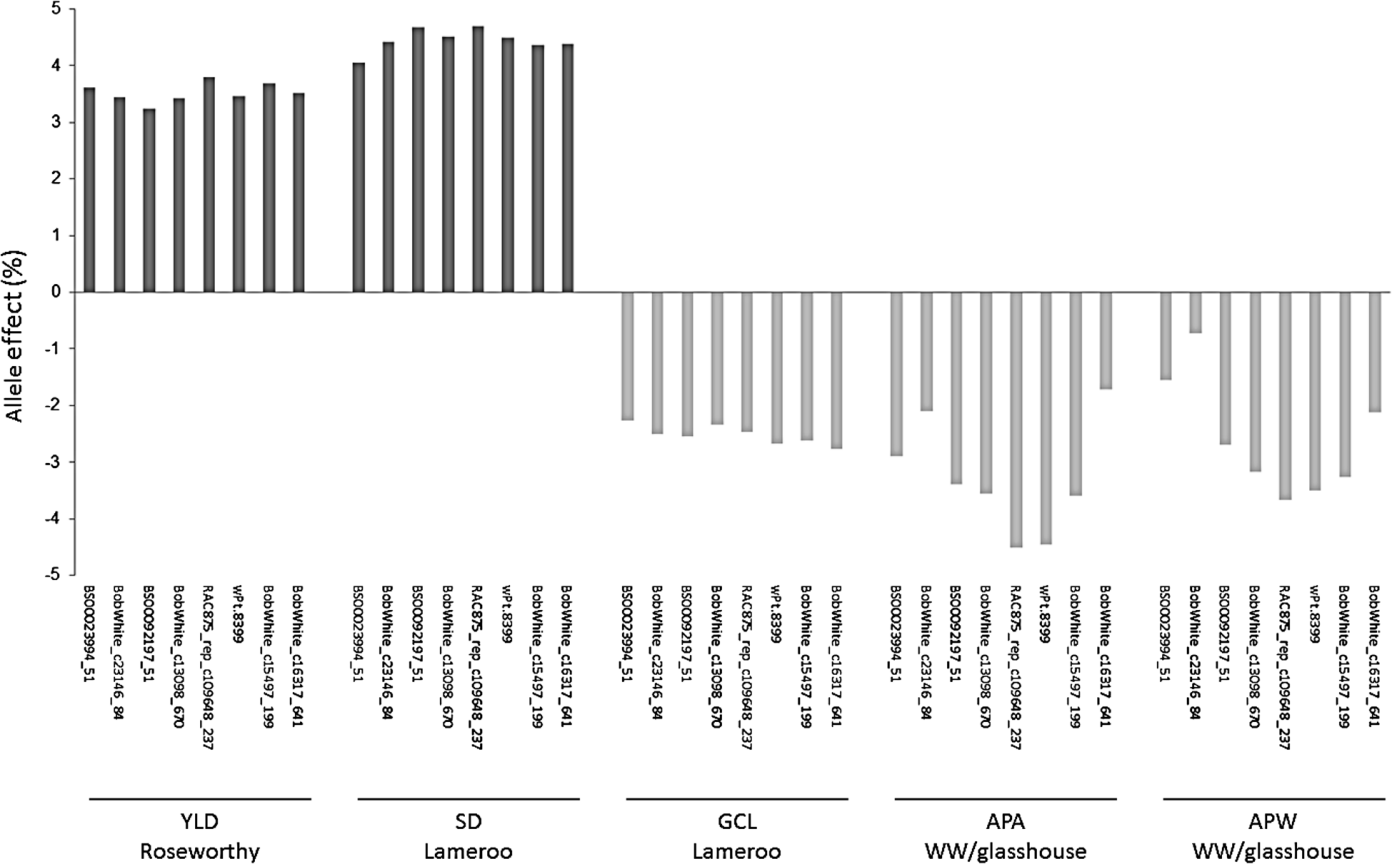
Allele effects of eight SNP covering the overlap among chromosome 7A1L QTL. Allele effects are presented as percentage relative to the trait mean for YLD in Roseworthy, SD and GCL in Lameroo and APW and APA under well-watered treatment in the glasshouse (WW). A positive effect indicates that the RAC875 allele increased the trait value (dark grey) while a negative effect indicates that the Kukri allele increased the trait value (light grey). The allele effects of all the loci are highly significant at *p* < 0.01

Some QTL affecting stomatal traits were also associated with yield on linkage group 7A1L (Table 3). In this region, the QTL *QSD.afl-7A* was detected for stomatal density in Lameroo and three QTL, *QAPL.afr-7A*, *QGCL.afr-7A* and *QYLD.afr-7A*, for aperture and guard cell lengths and yield in the Roseworthy field trial. These loci carried RAC875 as a positive allele increasing these traits. To further investigate this chromosomal region, the magnitudes and directions of allelic effects at the eight common loci in the QTL peak for each trait were statistically tested. A highly significant effect was found for yield and stomatal density, with the favorable allele coming from RAC875 in both the Roseworthy and Lameroo experiments. Allelic effects for GCL in Lameroo and APW and APA in the glasshouse were significant and negative, indicating that the Kukri allele increased the traits value (Fig. 3).

## Discussion

Stomatal conductance, photosynthesis, respiration and water transport from soil through roots, stem and leaves are coupled mechanisms in a soil-plant-atmosphere-continuum [19]. Here we considered only the stomata, the gates that control water exit and CO_2_ entry in the above ground portion of growing wheat plants. The major aim of this study was to identify QTL for stomatal size and density in relation to yield in wheat under drought stress. We found a total of 18 QTL for stomatal traits and three significant QTL for yield (Table 2). One of these yield QTL, *QYLD.afr-7A*, overlapped on the interval 75.3–101.4 cM of chromosome 7AL1 with QTL for stomatal traits, six identified in Roseworthy and two QTL under controlled-conditions.

### The drought tolerant cultivar RAC875 has small and numerous stomata

The RAC875 parental line has been used as a genetic source for breeding for drought tolerance in the Southern Australian environment, which is characterized by winter rainfall, terminal drought and heat stresses and shallow soils with low water storage. RAC875 shows high tolerance to drought and high air temperatures during grain filling relative to other cultivars. For example during the severe drought over the 2006 season where average yield was 0.8 t/ha, RAC875’s yield was consistently higher by 122 % of the site means [20].

Maintenance of stomatal conductance is critical to optimum growth rate and yield [19]. In the present study, RAC875 showed a higher frequency of stomata, and smaller aperture and guard cell size in flag leaves than Kukri under field and controlled conditions. By contrast with drought tolerant Arabidopsis mutants that showed large stomata in small density [13], Baloch et al. [21] reported similar results than ours. Drought tolerant cultivars of spring wheat produced smaller stomata, decreased stomatal conductance and increased relative water content under 65 % water stress. Similarly, stomatal density increased while stomatal size decreased with increasing water deficit in the grass species *Leymus chinensis* [9, 22].

It has been proposed that small guard cells may cause stomata to remain open and keep a balance between carbon gain from photosynthesis and the prevention of excessive water loss via transpiration in response to drought [22]. RAC875 has been previously reported to have a smaller leaf area and equal agronomic water use efficiency (6.5 g/l) compared to Kukri under cyclic drought [23] and show conservative strategy with a moderate osmotic adjustment in plant tissues, low stomatal conductance, low transpiration in response to vapour pressure deficits and a high sensitivity to a decreased fraction of transpirable soil water [23, 24]. RAC875 has a limited root hydraulic conductivity and small metaxylem elements [24]. These traits and small stomata in a high density would together enable RAC875 to keep C fixation active in the leaves and reduce plant water demand from the soil, thus conserving water for the critical phase of grain filling later in the season.

### Genetic determination of stomatal size and density

Stomatal aperture length is the linear distance between the junctions of the guard cells at each end of stomata [25]. While the width of the aperture and of the guard cells can increase or decrease quickly in response to small variations of the environmental conditions during the day, the length remains the same and determines the maximum potential of aperture size [26]. We observed highly significant differences among DH lines for aperture and guard cell lengths, but less so for width, which indicated that the leaf impression was an accurate and reproducible method to measure genetic variation in the maximum potential opening of stomatal pores.

We observed significant negative correlations between stomatal density and size measured as length, width and the area of guard cells and stomatal apertures. We also detected co-located QTL for these traits on chromosome arm 5BS under controlled conditions and on linkage group 7A1L where the RAC875 allele reduced stomatal aperture and guard cell length and increased stomatal density in field conditions. Such QTL affecting both traits could either be due to two loci in linkage, one controlling stomatal density and one controlling stomatal size, or to pleiotropic effects of a single locus that would affect stomata number and stomatal cell growth. Little is known about gene controlling stomata cell size as most developmental studies focused on stomatal cell fate specification and division (reviewed in [15]). Our study in wheat could be the start point of the discovery of genes controlling stomatal size.

Khasaei et al. [27] found similar results in wheat lines with different ploidy levels and reported a compensatory relationship between stomatal density and size to maintain an approximately equal total pore area on the leaf surface. Plants also reduce their leaf size in response to drought in order to maintain their hydraulic balance, stomatal opening and stomatal conductance [13]. This suggests there might also be compensation mechanisms between stomatal density and size and leaf area to achieve a whole plant stomatal area. Future experiments will also include measurements of the flag leaf size and whole plant above ground surface area to assess whether such mechanisms are involved in the function of the QTL described here.

### Stable QTL for stomatal traits across environments

Our experiment under controlled conditions aimed to validate QTL found in the field. The population was therefore narrowed down to focus on lines segregating for the QTL found in field trials. However, a small population size might lead to an underestimation of QTL numbers, overestimation of QTL effects, and failure to quantify QTL interactions [28, 29]. One strategy to reduce the effects of a small population size on QTL mapping is to use the genotypic information of recombinant lines at the tails of the phenotypic trait distribution, and use resampling techniques such as a permutation test to obtain unbiased estimates of QTL effects [28–31]. In the present study, we could identify a reliable threshold and detect significant QTL in the glasshouse experiment by using 55 recombinant plants from the 146 DH lines previously studied in the field along with a 5000 permutations test.

This method enabled us to identify QTL for stomatal traits in field trials that were also expressed under controlled conditions on the chromosome arms 1AS, 1BL, 2BL and 7A1L (Table 3). This means that these QTL control stomatal features at a single plant level, independent of the crop canopy architecture of wheat plants grown in field plots. This is an important finding as it will enable us to conduct detailed physiological studies of the QTL effects under controlled conditions where gas exchange and water use efficiency can be more easily and reliably measured than in the field. It is also worth noting that the field QTL overlap glasshouse QTL under specific watering conditions: the 1A QTL and 7A1 QTL from field trials overlap QTL found under well-watered conditions, while the 1B and 2B QTL collocate with QTL identified under drought. This information might indicate a specificity of mechanism of these QTL toward water availability.

### Co- location of QTL for stomatal traits and yield

As previously reported in wheat [32, 33], our study showed weak and non-significant correlation coefficients between yield and stomatal traits under field conditions. However, Khasaei et al. [33] showed by using a sequential path analysis that the effects of stomatal traits on yield operate indirectly through other traits like gas exchange and water use efficiency. This might be the case of the QTL that control both stomatal traits and yield.

Our study showed that several QTL for stomatal traits and yield are located on a nearly 40 cM sub-centromeric region of the linkage group 7A1L (Table 3). Bennett et al. [34] also detected QTL for kernel number per spike, normalized difference vegetation index, yield and harvest index in a similar region on chromosome 7AL in the RAC875/Kukri population. A close look at the markers where the QTL *QSD.afl-7A*, *QAPL.afr-7A*, *QGCL.afr-7A* and *QYLD.afr-7A* overlap showed significant effects of a 11.3 cM region where the RAC875 allele increased yield and stomatal density, while decreasing guard cell length and aperture area and width (Fig. 3). This region would be a useful target for selection in breeding programs. As the interval is still large, fine genetic mapping will be necessary to demonstrate that these QTL are a unique locus with pleiotropic effects. The region flanked by BS00023994_51 and BobWhite_c16317_641 markers include a total of 528 SNP polymorphic in RAC875/Kukri population that could be used to generate a high resolution genetic map using the 3000 recombinant inbred lines available for this cross [20].

Overlapping QTL for traits evaluated in the field and the glasshouse were also found on a 63.6 cM region on the long arm of chromosome 1B with a positive additive effect of the RAC875 allele for all these loci (Table 3). This large region might cover two QTL adjacent to each other, the first one in the interval 108.3–116.5 controlling aperture width, guard cell length and aperture in the glasshouse, and a second QTL controlling guard cell length under drought in the glasshouse and guard cell aperture in Lameroo field trial. This region also coincides with two co-located QTL for kernel number per spike and yield identified by Bennett et al. [35] in the same population grown in different water-limited environments. Fine mapping will be required to elucidate such a large 63.6 cM interval and validate the co-location of those QTL.

Finally, the QTL for yield in Lameroo field trial and for stomatal density in Roseworthy field trial on chromosome arm 4AS overlapped with QTL for yield identified in a previous study [35] on the same population. However the study by Bennett et al. showed an opposite allelic effect of RAC875 compared to this study. Moreover these QTL were not co-located entirely and could be independent QTL.

## Conclusions

Because stomatal development is only a component of drought tolerance mechanisms in plants that eventually translate to yield, it is not surprising that no phenotypic correlation were found between stomatal traits and yield. However the QTL co-locations found in our study suggested that stomatal traits could be an underlying mechanism increasing yield at specific loci. This finding could help accelerating the positional cloning of yield QTL. Firstly, stomatal measurement could be used as a “proxy” trait for selecting yield QTL. The method presented here is inexpensive and requires only a small number of plants, unlike a field grown plot for yield measurement. A QTL controlling both yield and stomatal traits could easily be tracked in large recombinant populations by evaluating the stomatal traits under controlled conditions to decrease the G × E effects that usually impair yield evaluation in field trials. Secondly, the specific effect of a QTL on stomata would help identifying the candidate genes at the locus and clone yield QTL. This would assist wheat breeders to select traits that maintain yield under drought conditions more efficiently.

## Methods

### Mapping population

A doubled-haploid population derived from a cross between RAC875 (RAC655/3/Sr21/4*Lance//4*Bayonet) and Kukri (76ECN44/76ECN36//Madden/6*RAC177) spring type bread wheat cultivars. The RAC875 is a breeding line that has previously shown a relatively stable yield in water-limited conditions, while Kukri is a locally adapted variety that has significantly reduced grain yield under the same conditions [20, 23]. To minimise the confounding impact of phenology, 146 lines flowering within a 2 weeks window were selected for planting [36]. Distribution for Zadoks’ score in Lameroo 2012 is shown in supplemental data (Additional file 3: Figure S2).

### Field trials

The DH lines and their parents were grown under rain-fed conditions in Lameroo in 2012 (35° 33′ S; 140° 52′ E, with average annual rainfall from onsite weather station: 382.1 mm) and Roseworthy in 2013 (34° 53′ S; 138° 69′ E, with average annual rainfall from onsite weather station: 440.3 mm), South Australia. According to the Bureau of Meteorology, 2012 was a dry year in South Australia with 77 % of average rainfall (http://www.bom.gov.au/climate/current/annual/sa/archive/2012.summary.shtml). 2013 was the warmest year on record for South Australia with rainfall as a whole near average (http://www.bom.gov.au/climate/current/annual/sa/archive/2013.summary.shtml).

Lines were arranged in partially replicated (30 %) spatial design [36, 37] in Lameroo and randomized complete block design with two replications in Roseworthy. Fertiliser and herbicide application and management regime for each site followed best local practice. Grain yield data (YLD, t/ha) was collected from field plots 2.25 m wide and 3.5 m in length, and constituted of four rows. The fully expanded flag leaf of the main tiller of two plants per line in the middle of each plot at anthesis (Zadoks’ scale 69) [38] was used for leaf imprinting.

### Growth conditions in glasshouse

To reduce the impact of environmental variations on stomatal behaviour, a drought stress experiment of six weeks (Zadoks’ scale 31–49) was conducted between mid of April and June 2014 under controlled conditions at The Plant Accelerator glasshouse facilities of The University of Adelaide (Urrbrae, South Australia, 34° 58′S; 138° 38′E). Out of 146 RILs, a subset of 55 lines were selected as a preliminary population for QTL mapping [39, 40] based on their recombination on chromosome 7A where QTL were first identified for yield [34, 35] and on chromosomes with QTL for stomatal traits in field grown wheat plants (present study). This subset was grown under well-watered and stable drought treatments using a randomized complete block design with two blocks, where each line was replicated three times per block. Control and drought treatments of each line were placed next to each other.

Single plants were grown in 2.5 L plastic pots filled with 2.4 kg of soil (50 % coco-peat, 50 % clay-loam). Three seeds per pot were sown and the seedlings thinned to one plant per pot at the three-leaf stage. Plants were grown for two weeks in a regular glasshouse with manual watering to allow optimal germination and early growth. Thereafter, pots were weighed and watered every second day to 40 % gravimetric water content (-0.185 MPa soil water potential) for the well-watered treatment and 16 % (-0.5 MPa soil water potential) for the stress treatment. The experiments were conducted under natural lighting with the temperature in the greenhouse ranging from 15 °C (night) to 22 °C (day).

### Leaf imprinting

The impression approach was used to determine leaf stomatal traits [9] under field and glasshouse conditions. Flag leaves on the main tiller of two plants per plot that were fully expanded and fully exposed to the sun were collected at mid-day during sunny clear days to obtain measurements in steady-state conditions [14]. A non-destructive leaf impressions were made by applying high viscosity (>21 mm^2^/s at 40 °C) cyanoacrylate adhesive (Selleys Auto Fix Supaglue, Australia) on intact leaves and peeling right away the adhesive without moving the leaf or the plant to the lab.

A pilot study using 20 RAC875/Kukri DH lines had shown that the stomatal traits were highly positively correlated between the adaxial and abaxial sides of the leaf in this genetic material. This was supported by a previous study [41] that found that wheat leaves had similar numbers of stomata on each leaf surface. Due to the workload involved in sampling many lines, and to limit any variations due to differences in sampling time, impressions were taken of only the adaxial (upper) side of flag leaves for the entire population. The adaxial surface was chosen measurements on the whole population because it showed the highest genetic variation for stomatal traits in the pilot experiment.

The glue was applied on the adaxial surface of the flag leaf at the mid-point between the central vein and the leaf margin, and half way along the long axis of the leaf. The thin imprints (area approximately 25 mm × 17 mm) were peeled off from the leaf surface and immediately mounted on a glass slide (75 mm x 25 mm). Images of the stomata were observed using the differential interference contrast techniques with a Leica microscope (Leica AS LMD laser dissection, Leica Microsystems, Australia). After focusing, three pictures of each leaf were taken at 20 times magnification. Subsequent image analyses were performed using ImageJ software available at: http://imagej.nih.gov/ij/, 1997–2014.

The number of stomata were counted in 0.45 mm^2^ area per picture to determine stomatal density (SD, n/mm^2^). The leaf stomatal index (SI, %) was estimated by counting total number of epidermal cells (EC, n/mm^2^) per picture and applying the formula: [SD/(SD + EC)]*100 [42]. For the determination of stomatal size related traits (Additional file 4: Figure S3), five stomata per picture were measured for length (APL, μm), width (APW, μm) and area (APA, μm^2^) of aperture pore and length (GCL, μm), width (GCW, μm) and area (GCA, μm^2^) of guard cells [8, 17].

### Statistical analysis

The variance components and the best linear unbiased predictors for each line and average of the traits were calculated using PROC GLM in SAS v.6. Broad sense heritability (*h*^*2*^) was estimated from variance components according to Kearsey and Pooni [43]. Descriptive statistics and frequency distribution of the traits were calculated using SPSS v.10.0. Pearson correlation heat map of all the traits was obtained using GenStat v.10 available at: http://www.vsni.co.uk/products/genstat/.

### Genetic map

The first genetic map of the RAC875/Kukri population was constructed using 610 simple sequence repeats (SSR) and diversity arrays technology (DArT) markers [35]. The map has been enriched and re-constructed by incorporating 15,508 single nucleotide polymorphism (SNP) markers from high-throughput 90,000 gene-associated SNP iSelect Bead Chip array [44] as described in Mahjourimajd [45].

Briefly, a total number of 15,911 markers comprised of 235 SSR, 160 DArT, 15,508 SNP, 2 insertion site-based polymorphism and 6 gene-based markers were assembled into 26 linkage groups and assigned to 21 wheat chromosomes in the RAC875/Kukri mapping population. The total length of the genetic map is 2864 cM, containing 2356 unique loci with an average distance of 1.23 cM (min = 0.1 and max = 48.1 cM) between two markers. From the enriched SNP map, a ‘base map’ consisting of 1345 markers per cluster of co-segregated markers was used for QTL mapping.

### QTL mapping

QTL analysis was performed only for traits which showed significant variation among the DH lines. Initially, single marker analysis was performed for each trait to identify markers associated with variations. Further evaluation was carried out by composite interval mapping with a 15 cM window and a maximum of 15 marker cofactors per model using Windows QTL Cartographer version 2.0.

The plant response to drought can be confounded by environmental covariates which relate to differing plant phenology. In our study we removed the phenology differences first by selecting DH lines that flower within 2 weeks. The remaining phenology effect (as shown by the Zadok’score frequency distribution in Additional file 3: Figure S2) is then likely due to the *Photoperiod Ppd-B1* and *Ppd-D1* genes that still segregate in the RAC875/Kukri population. *Ppd-B1* and *Ppd-D1* genes regulate flowering time in response to photoperiod and have pleiotropic effects on plant growth and development [46]. To remove the effect of photoperiod genes on the traits, *Ppd-B1* and *Ppd-D1* markers [47] specified as cofactors and all the other markers as control to determine the genetic background in the CIM analysis. Tests were performed at 1 cM intervals by forward-backward stepwise regression (Model 6).

Genome wide, trait specific, threshold values (α = 0.05) of the likelihood ratio (LR) test statistic for declaring the presence of a QTL was estimated from a 1000–5000 permutations test by random sampling of phenotypic data [30, 31]. The phenotypic variation explained by a QTL (R^2^) conditioned by the composite interval mapping cofactors included in the model was calculated at the most likely QTL position. The additive effect of an allelic substitution at each QTL was also obtained. The LOD peak of each significant QTL was considered as the QTL location on the linkage map. To detect significant allelic effect for single markers at the chromosomal region of interest, Wald statistics were applied [48].

## Abbreviations

APA, aperture area; APL, aperture length; APW, aperture width; Chr, chromosome; cM, centiMorgan; DArT, diversity arrays technology; DH, doubled haploid; EC, epidermal cell; GCA, guard cell area; GCL, guard cell length; GCW, guard cell width; h^2^, heritability; LOD, log of odds; ns, non-significant; p, probability; PSII, photosystem II; QTL, quantitative trait loci; SD, stomatal density; SI, stomatal index; SNP, single nucleotide polymorphism; SSR, simple sequence repeat; STD, standard deviation; YLD, yield.

## Additional files

Additional file 1: Figure S1. Frequency distribution of phenotypes for stomatal size related traits and yield in the RAC875/Kukri DH lines based on means obtained over each experiment. a) Lameroo, b) Roseworthy, c) Well watered conditions in the glasshouse, d) Drought conditions in the glasshouse. Arrows indicate phenotypic values of RAC875 (R) and Kukri (K). (PPTX 264 kb) Additional file 2: Table S1. Mean square and probability of ANOVA to test significant differences among the lines for stomatal traits and yield under well-watered (WW) *versus* drought (D) in the glasshouse and Lameroo *versus* Roseworthy in field conditions. (XLSX 11 kb) Additional file 3: Figure S2. Frequency distribution of Zadok’score of the 146 RAC875/Kukri DH lines in Lameroo 2012 field trial. (PPTX 44 kb) Additional file 4: Figure S3. Morphological features of a single stomata. Arrows indicate aperture length (APL) and width (APW) and guard cell length (GCL) and width (GCW). Aperture area (APA) and guard cell area (GCL) were calculated by multiplying the length and width of the rectangle. (DOCX 261 kb)

## References

[1] Tester M, Langridge P (2010). Breeding technologies to Increase crop production in a changing world. Science.

[2] Reynolds M, Bonnett D, Chapman SC, Furbank RT, Manes Y, Mather DE, Parry MA (2011). Raising yield potential of wheat. I. Overview of a consortium approach and breeding strategies. J Exp Bot.

[3] Quiggin J. Drought, climate change and food prices in Australia. In. School of Economics and School of Political Science and International Studies, University of Queensland, St Lucia, QLD Australia; 2007.

[4] Volkmar S (1963). The translocation of C14-labelled photosynthetic products from the leaf to the ear in wheat. Physiol Plant.

[5] Verma V, Foulkes MJ, Worland AJ, Sylvester-Bradley R, Caligari PDS, Snape JW (2004). Mapping quantitative trait loci for flag leaf senescence as a yield determinant in winter wheat under optimal and drought-stressed environments. Euphytica.

[6] Blake NK, Lanning SP, Martin JM, Sherman JD, Talbert LE (2007). Relationship of flag leaf characteristics to economically important traits in two spring wheat crosses. Crop Sci.

[7] Biswal AK, Kohli A (2013). Cereal flag leaf adaptations for grain yield under drought: knowledge status and gaps. Mol Breed.

[8] Hetherington AM, Woodward FI (2003). The role of stomata in sensing and driving environmental change. Nature.

[9] Xu Z, Zhou G (2008). Responses of leaf stomatal density to water status and its relationship with photosynthesis in a grass. J Exp Bot.

[10] Ainsworth EA, Rogers A (2007). The response of photosynthesis and stomatal conductance to rising [CO2]: mechanisms and environmental interactions. Plant Cell Environ.

[11] Cattivelli L, Rizza F, Badeck FW, Mazzucotelli E, Mastrangelo AM, Francia E, Mare C, Tondelli A, Stanca AM (2008). Drought tolerance improvement in crop plants: An integrated view from breeding to genomics. Field Crops Res.

[12] Messmer R, Fracheboud Y, Banziger M, Vargas M, Stamp P, Ribaut JM (2009). Drought stress and tropical maize. QTL-by-environment interactions and stability of QTLs across environments for yield components and secondary traits. Theor Appl Genet.

[13] Doheny-Adams T, Hunt L, Franks PJ, Beerling DJ, Gray JE (2012). Genetic manipulation of stomatal density influences stomatal size, plant growth and tolerance to restricted water supply across a growth carbon dioxide gradient. Phil Trans Royal Society B-Biological Sciences.

[14] Dillen SY, Marron N, Koch B, Ceulemans R (2008). Genetic variation of stomatal traits and carbon isotope discrimination in two hybrid poplar families (Populus deltoides 'S9-2' x P. nigra 'Ghoy' and P. deltoides 'S9-2' x P. trichocarpa 'V24'). Ann Bot.

[15] Pillitteri LJ, Torii KU (2012). Mechanisms of stomatal development. Ann Rev Plant Biol.

[16] Gailing O, Langenfeld-Heyser R, Polle A, Finkeldey R (2008). Quantitative trait loci affecting stomatal density and growth in a Quercus robur progeny: implications for the adaptation to changing environments. Global Change Biol.

[17] Liu X, Mak M, Babla M, Wang F, Chen G, Veljanoski F, Wang G, Shabala S, Zhou M, Chen ZH (2014). Linking stomatal traits and expression of slow anion channel genes HvSLAH1 and HvSLAC1 with grain yield for increasing salinity tolerance in barley. Front Plant Sci.

[18] Laza MRC, Kondo M, Ideta O, Barlaan E, Imbe T (2010). Quantitative trait loci for stomatal density and size in lowland rice. Euphytica.

[19] Roche D (2015). Stomatal conductance is essential for higher yield potential of C-3 crops. Crit Rev Plant Sci.

[20] Fleury D, Jefferies S, Kuchel H, Langridge P (2010). Genetic and genomic tools to improve drought tolerance in wheat. J Exp Bot.

[21] Baloch MJ, Dunwell J, Khan NU, Jatoi WA, Khakhwani AA, Vessar NF, Gul S (2013). Morpho-physiological Characterization of Spring Wheat Genotypes under Drought Stress. Int J Agric Biol.

[22] Spence RD, Wu H, Sharpe PJH, Clark KG (1986). Water-Stress Effects on Guard-Cell Anatomy and the Mechanical Advantage of the Epidermal-Cells. Plant Cell Environ.

[23] Izanloo A. Evaluation of physiological traits and identification of QTLs for drought tolerance in hexaploid wheat (Triticum aestivum L.). Thesis University of Adelaide, Adelaide, SA, Australia. 2008.

[24] Schoppach R, Wauthelet D, Jeanguenin L, Sadok W (2014). Conservative water use under high evaporative demand associated with smaller root metaxylem and limited transmembrane water transport in wheat. Funct Plant Biol.

[25] Zheng YP, Xu M, Hou RX, Shen RC, Qiu S, Ouyang Z (2013). Effects of experimental warming on stomatal traits in leaves of maize (Zea may L.). Ecol Evol.

[26] Beaulieu JM, Leitch IJ, Patel S, Pendharkar A, Knight CA (2008). Genome size is a strong predictor of cell size and stomatal density in angiosperms. New Phytol.

[27] Soares-Cordeiro AS, Driscoll SP, Pellny TK, Olmos E, Arrabaca MC, Foyer CH (2009). Variations in the dorso-ventral organization of leaf structure and Kranz anatomy coordinate the control of photosynthesis and associated signalling at the whole leaf level in monocotyledonous species. Plant Cell Environ.

[28] Khazaei H, Monneveux P, Shao HB, Mohammady S (2010). Variation for stomatal characteristics and water use efficiency among diploid, tetraploid and hexaploid Iranian wheat landraces. Genet Resour Crop Evol.

[29] Beavis W, P AH (1998). QTL analyses: power, precision, and accuracy. Molecular dissection of complex traits.

[30] Vales MI, Schon CC, Capettini F, Chen XM, Corey AE, Mather DE, Mundt CC, Richardson KL, Sandoval-Islas JS, Utz HF (2005). Effect of population size on the estimation of QTL: a test using resistance to barley stripe rust. Theor Appl Genet.

[31] Churchill GA, Doerge RW (1994). Empirical threshold values for quantitative trait Mapping. Genetics.

[32] Doerge RW, Churchill GA (1996). Permutation tests for multiple loci affecting a quantitative character. Genetics.

[33] Maghsoudi K, Maghsoudi MA (2008). Analysis of the effects of stomatal frequency and size on transpiration and yield of wheat (Triticum aestivum L). American-Eurasian J Agric Environ Sci.

[34] Khazaie H, Mohammady S, Monneveux P, Stoddard F (2011). The determination of direct and indirect effects of carbon isotope discrimination (Delta), stomatal characteristics and water use efficiency on grain yield in wheat using sequential path analysis. Aust J Crop Sci.

[35] Bennett D, Izanloo A, Reynolds M, Kuchel H, Langridge P, Schnurbusch T (2012). Genetic dissection of grain yield and physical grain quality in bread wheat (Triticum aestivum L.) under water-limited environments. Theor Appl Genet.

[36] Bennett D, Reynolds M, Mullan D, Izanloo A, Kuchel H, Langridge P, Schnurbusch T (2012). Detection of two major grain yield QTL in bread wheat (Triticum aestivum L.) under heat, drought and high yield potential environments. Theor Appl Genet.

[37] Brownie C, Bowman D, Burton J (1993). Estimating spatial variation in analysis of data from yield trials: A comparison of methods. Agron J.

[38] Qiao CG, Basford KE, DeLacy IH, Cooper M (2000). Evaluation of experimental designs and spatial analyses in wheat breeding trials. Theor Appl Genet.

[39] Zadoks JC, Chang TT, Konzak CF (1974). A decimal code for the growth stages of cereals. Weed Res.

[40] Mohan M, Nair S, Bhagwat A, Krishna TG, Yano M, Bhatia CR, Sasaki T (1997). Genome mapping, molecular markers and marker-assisted selection in crop plants. Mol Breed.

[41] Collard BCY, Jahufer MZZ, Brouwer JB, Pang ECK (2005). An introduction to markers, quantitative trait loci (QTL) mapping and marker-assisted selection for crop improvement: The basic concepts. Euphytica.

[42] O'Carrigan A, Babla M, Wang FF, Liu XH, Mak M, Thomas R, Bellotti B, Chen ZH (2014). Analysis of gas exchange, stomatal behaviour and micronutrients uncovers dynamic response and adaptation of tomato plants to monochromatic light treatments. Plant Physiol Biochem.

[43] Kearsey MJ, Pooni HS. The genetical analysis of quantitative traits. Stanley Thornes (Publishers) Ltd, Cheltenham, UK; 1998.

[44] Wang S, Wong D, Forrest K, Allen A, Chao S, Huang BE, Maccaferri M, Salvi S, Milner SG, Cattivelli L (2014). Characterization of polyploid wheat genomic diversity using a high-density 90,000 single nucleotide polymorphism array. Plant Biotechnol J.

[45] Mahjourimajd S: Dissecting genetic variation for nitrogen use efficiency in wheat. Thesis. The University of Adelaide, Adelaide, SA, Australia. 2015.

[46] Cockram J, Jones H, Leigh FJ, O’Sullivan D, Powell W, Laurie DA, Greenland AJ (2007). Control of flowering time in temperate cereals: genes, domestication, and sustainable productivity. J Exp Bot.

[47] Beales J, Turner A, GriYths S, Snape JW, Laurie DA (2007). A Pseudo-Response Regulator is misexpressed in the photoperiod insensitive Ppd-D1a mutant of wheat (Triticum aestivum L.). Theor Appl Genet.

[48] Kenward MG, Roger JH (1997). Small sample inference for fixed effects from restricted maximum likelihood. Biometrics.

