# Supplementary material for: Genetic association of stomatal traits and yield in wheat grown in low rainfall environments
Source: BMC Plant Biol. 2016 Jul 4;16:150. doi: 10.1186/s12870-016-0838-9 (PMC4932692; doi:10.1186/s12870-016-0838-9)
Supplement: Additional file 1: Figure S1. — Frequency distribution of phenotypes for stomatal size related traits and yield in the RAC875/Kukri DH lines based on means obtained over each experiment. a) Lameroo, b) Roseworthy, c) Well watered conditions in the glasshouse, d) Drought conditions in the glasshouse. Arrows indicate phenotypic values of RAC875 (R) and Kukri (K). (PPTX 264 kb) [file 12870_2016_838_MOESM1_ESM.pptx]

## Slide 1
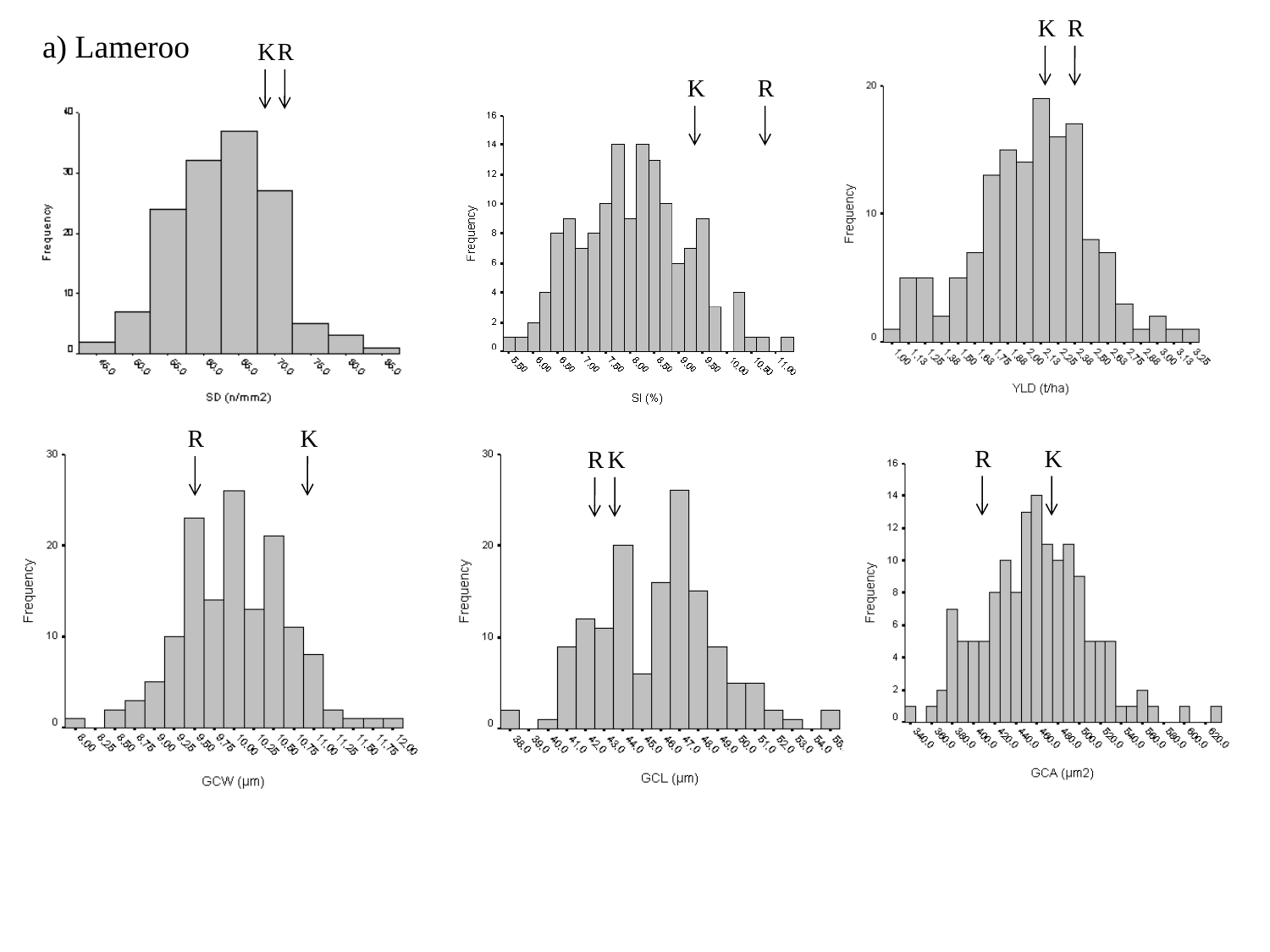

K
R
a) Lameroo
K
R
K
R
K
R
R
K
K
R

## Slide 2
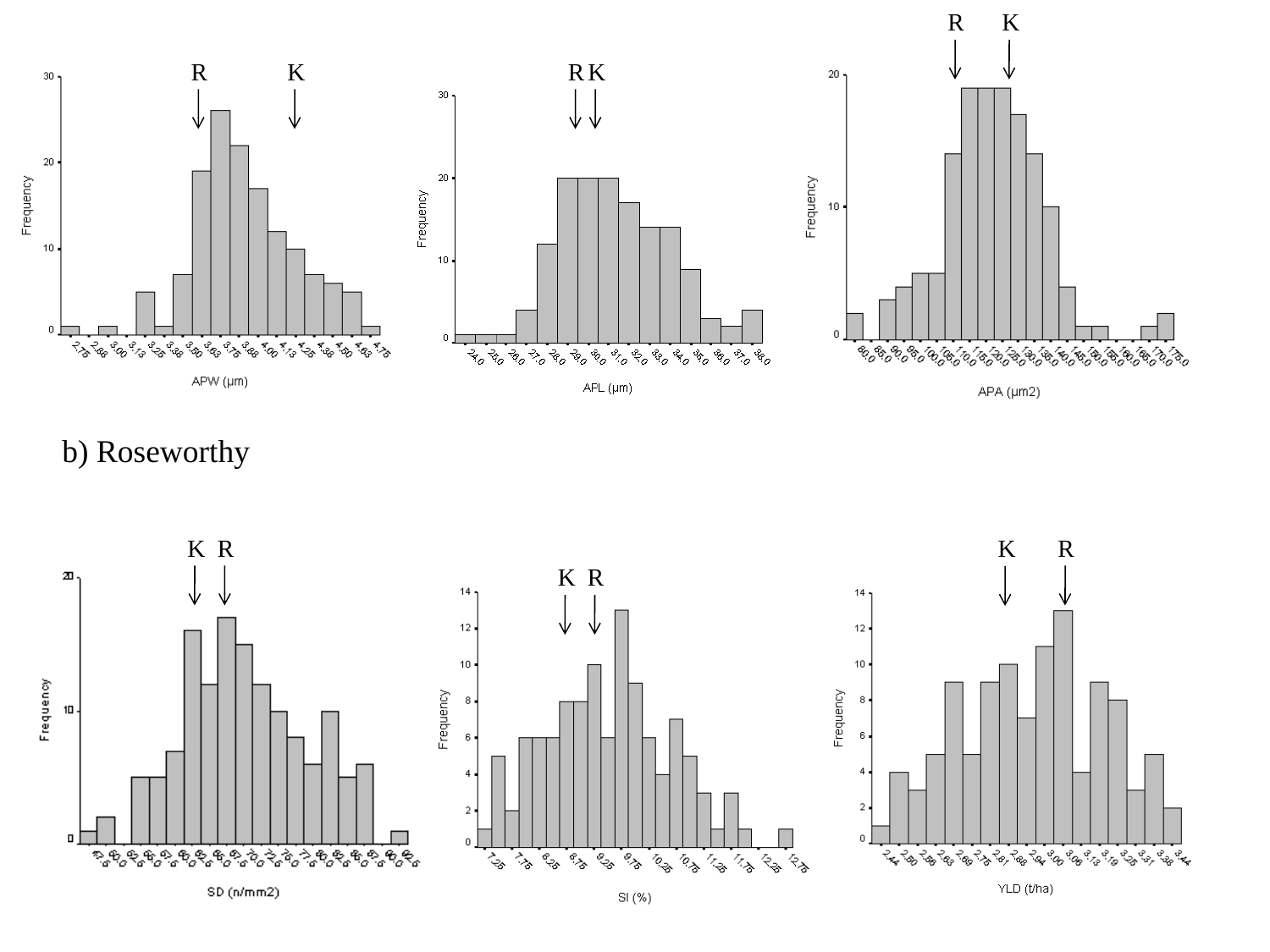

R
K
R
K
R
K
b) Roseworthy
K
R
R
K
R
K

## Slide 3
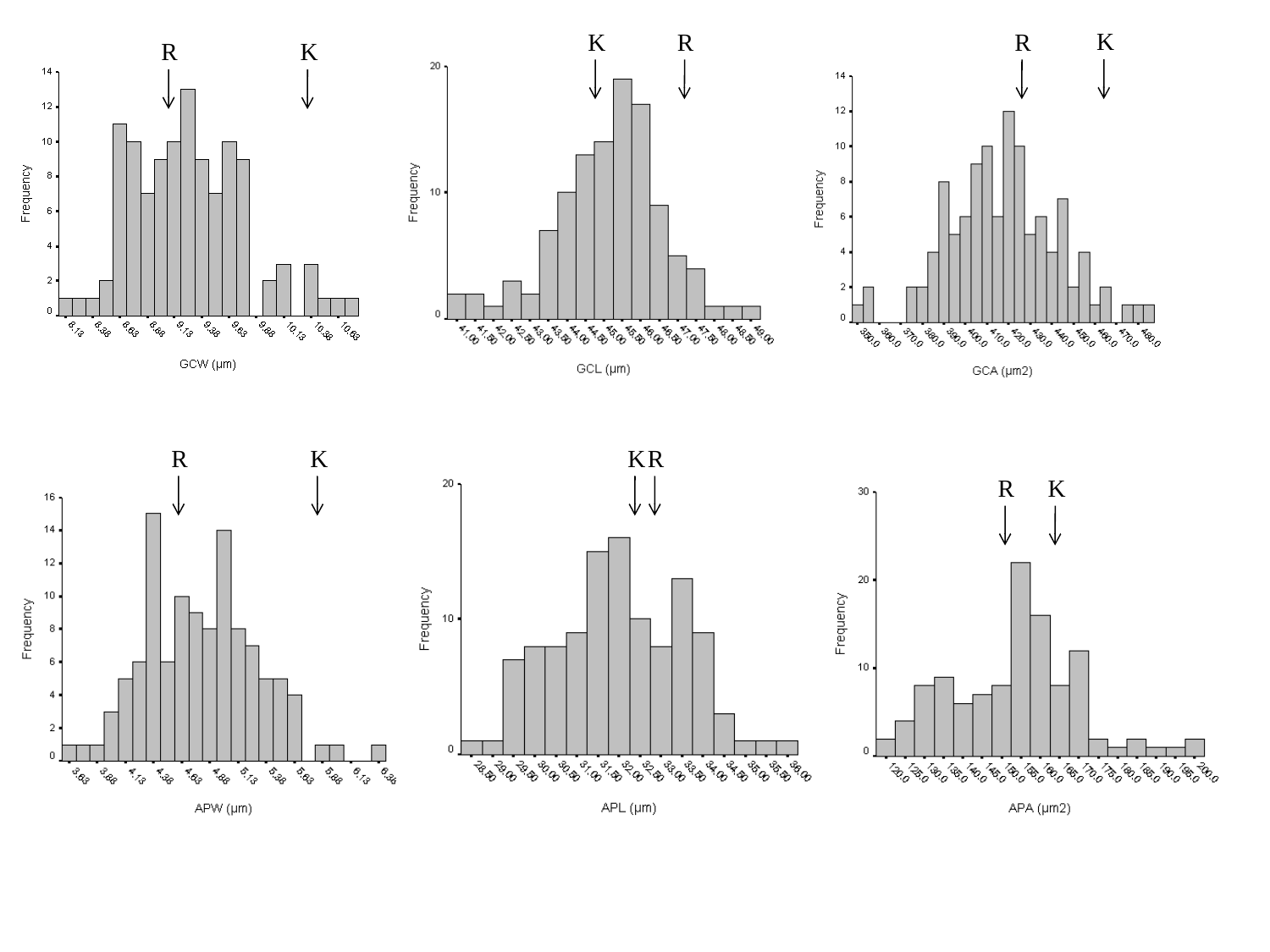

K
K
R
R
R
K
R
K
K
R
R
K

## Slide 4
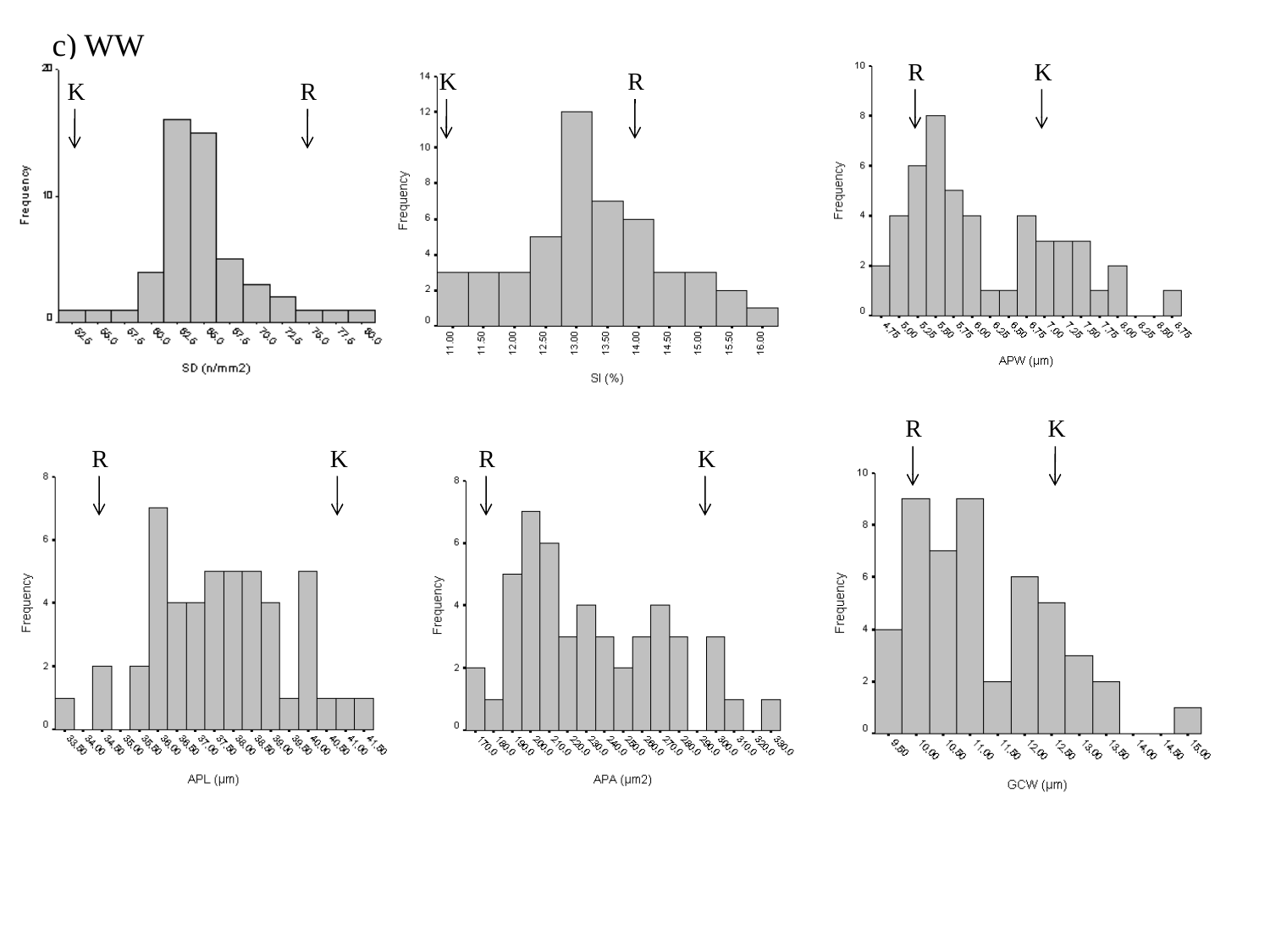

c) WW
R
K
R
K
K
R
R
K
R
K
R
K

## Slide 5
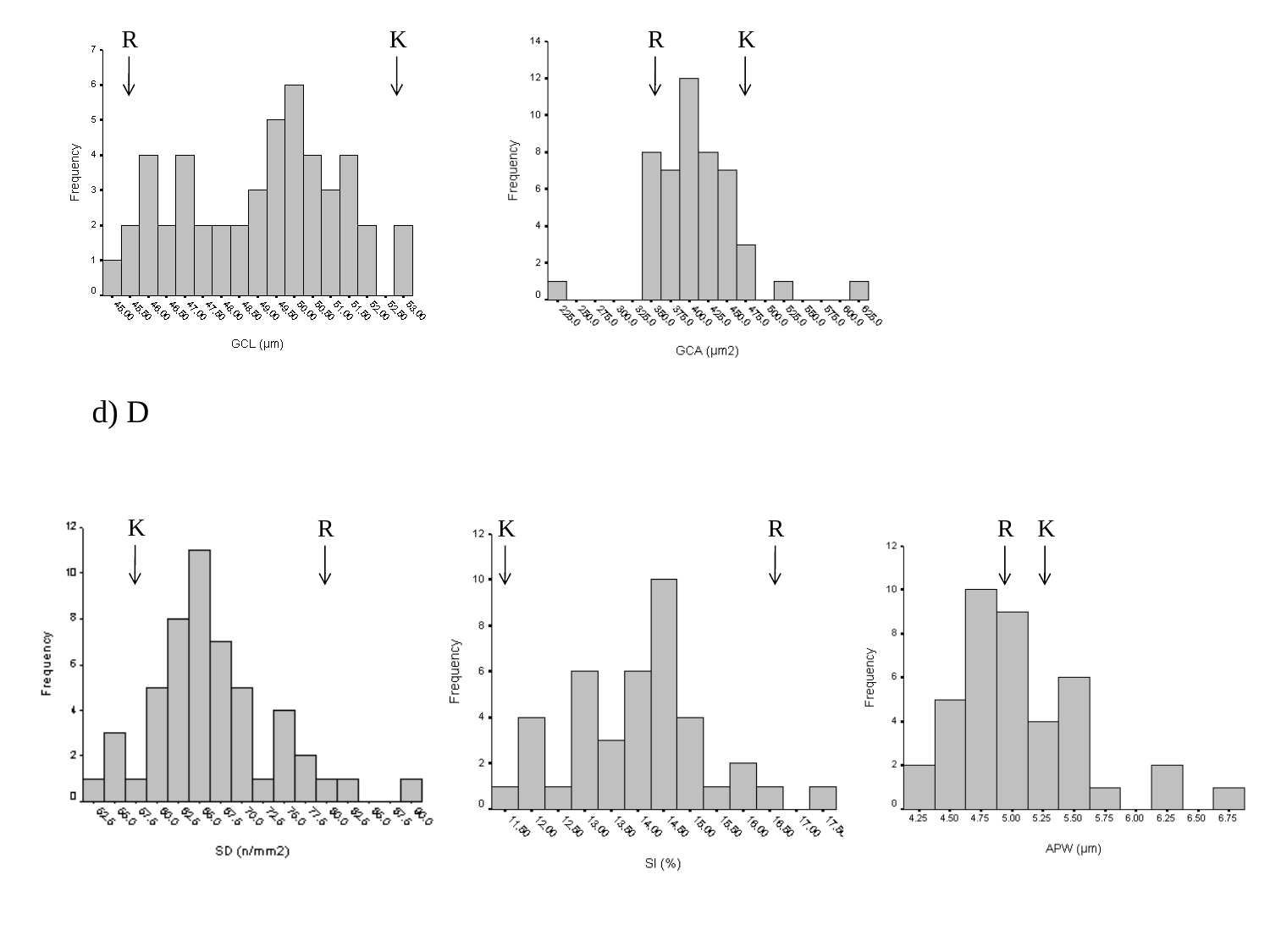

R
K
R
K
d) D
K
R
K
R
R
K

## Slide 6
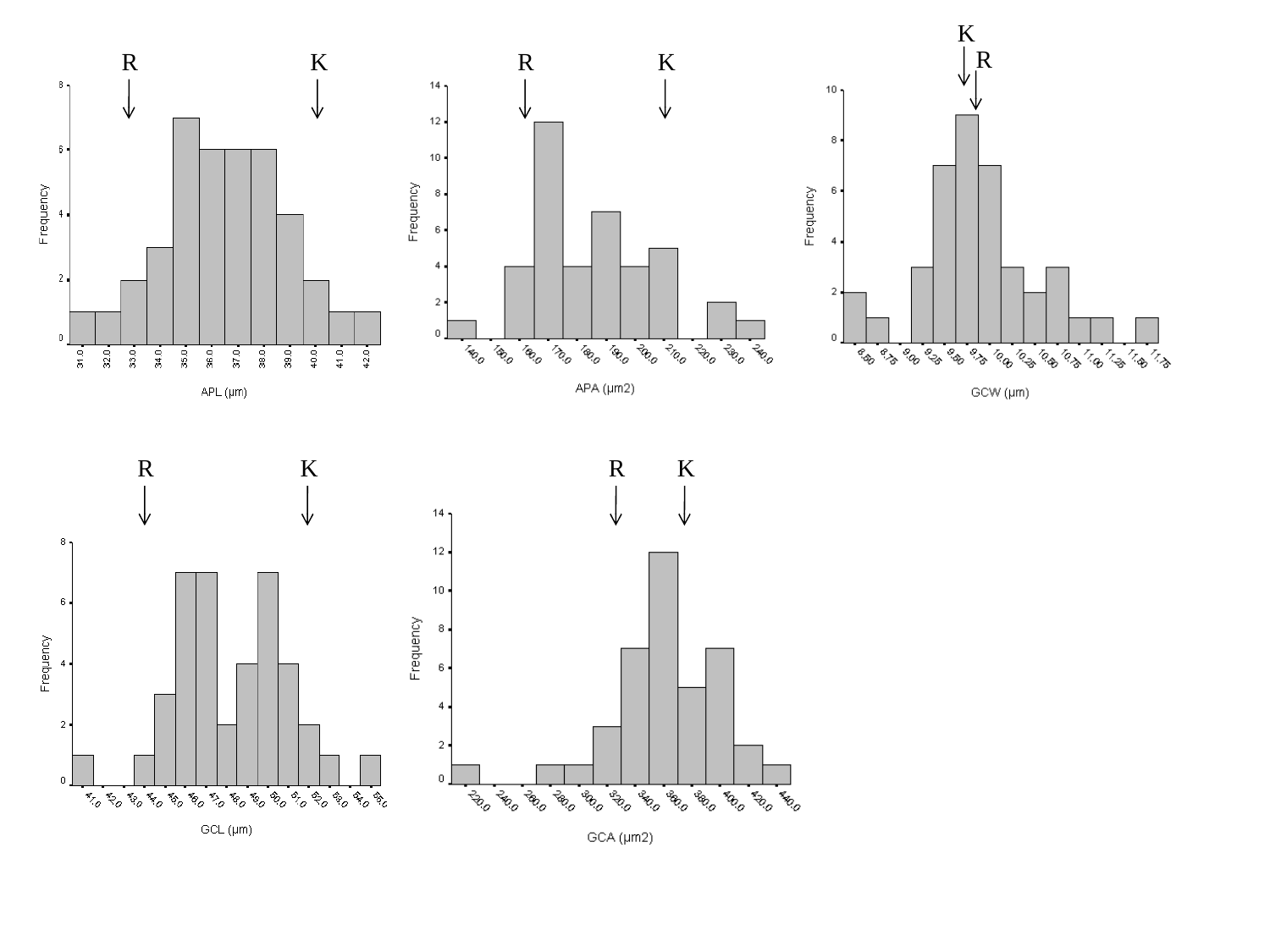

K
K
R
K
R
R
K
R
K
R
