# Supplementary figures and images for: Genetic association of stomatal traits and yield in wheat grown in low rainfall environments
Source: BMC Plant Biol. 2016 Jul 4;16:150. doi: 10.1186/s12870-016-0838-9 (PMC4932692; doi:10.1186/s12870-016-0838-9)

## Slide 1
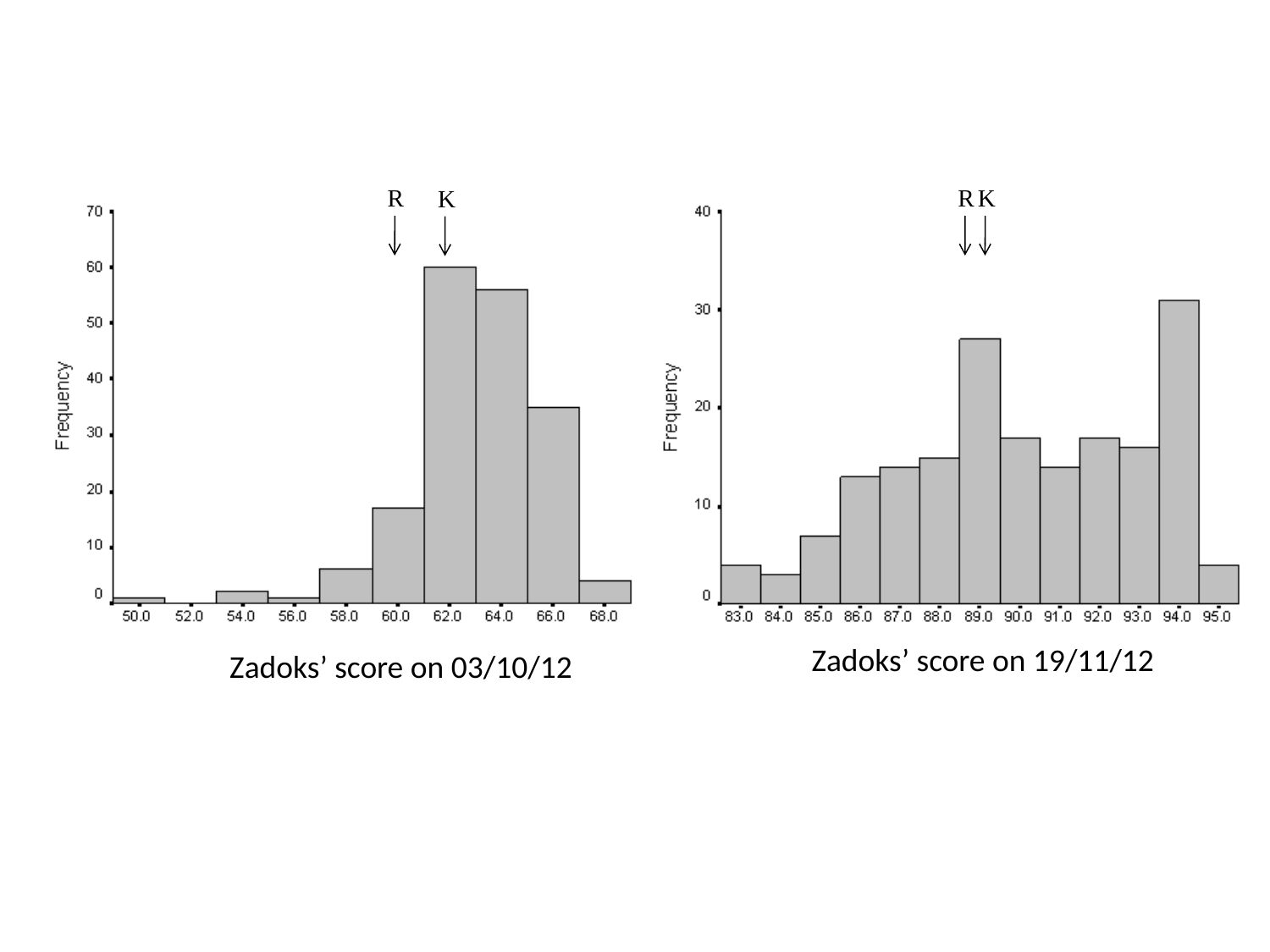

R
K
R
K
Zadoks’ score on 19/11/12
 Zadoks’ score on 03/10/12

Supplement: Additional file 3: Figure S2. — Frequency distribution of Zadok’score of the 146 RAC875/Kukri DH lines in Lameroo 2012 field trial. (PPTX 44 kb) [file 12870_2016_838_MOESM3_ESM.pptx]

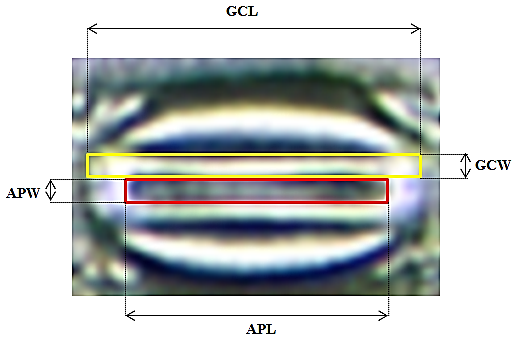

Supplement: Additional file 4: Figure S3. — Morphological features of a single stomata. Arrows indicate aperture length (APL) and width (APW) and guard cell length (GCL) and width (GCW). Aperture area (APA) and guard cell area (GCL) were calculated by multiplying the length and width of the rectangle. (DOCX 261 kb) [file 12870_2016_838_MOESM4_ESM.docx]
